# Is atopy a risk indicator of chronic obstructive pulmonary disease in dairy farmers?

**DOI:** 10.1186/s12931-019-1082-2

**Published:** 2019-06-17

**Authors:** Matthieu Veil-Picard, Thibaud Soumagne, Rechana Vongthilath, Isabella Annesi-Maesano, Alicia Guillien, Lucie Laurent, Pascal Andujar, Nicolas Roche, Stephane Jouneau, Benoit Cypriani, Jean-Jacques Laplante, Bruno Degano, Jean-Charles Dalphin

**Affiliations:** 1 Service de Pneumologie, Oncologie thoracique et Allergologie respiratoire, 3 Boulevard Fleming, 25000 Besancon, France; 2 Epidemiology of Allergic and Respiratory Diseases Department, IPLESP, INSERM and Sorbonne Université, 75012 Paris, France; 3 Equipe d’Epidémiologie Environnementale, Institute for Advanced Biosciences, Centre de Recherche UGA, INSERM U1209, CNRS UMR, 5309 Grenoble, France; 4 Université Paris-Est Créteil, Faculté de Médecine, Créteil, France; 5 Centre hospitalier intercommunal de Créteil, Service de Pathologie Professionnelle et de l’Environnement, Créteil, France; 6 Service de Pneumologie et Soins Intensifs Respiratoires, Groupe Hospitalier Cochin, Site Val de Grâce, AP-HP et Université Paris Descartes (EA2511), Sorbonne-Paris-Cité, Paris, France; 7 Service de Pneumologie, CHU de, Rennes, France; 8 UMR1085, IRSET, Université de Rennes 1, Rennes, France; 9 Laboratoire de biochimie CHRU de, Besançon, France; 10 Mutualité Sociale Agricole, Besançon, France; 11 Service Hospitalier Universitaire Pneumologie Physiologie, Pôle Thorax et Vaisseaux, CHU Alpes, Grenoble, France; 12 Université Grenoble Alpes and INSERM U1042, Grenoble, France; 13 UMR/CNRS 6249 Chrono-Environnement, Université de Franche-Comté, Besançon, France

**Keywords:** Atopy, Chronic obstructive pulmonary disease, Specific IgE, Farmers, Occupational exposure, Matthieu Veil-Picard and Thibaud Soumagne contributed equally to the work.

## Abstract

**Abstract:**

Allergic mechanisms related to environmental and occupational exposure have been suggested to contribute to the development of chronic obstructive pulmonary disease (COPD).

**Objectives:**

To investigate the relationships between atopy markers, persistent airflow limitation (PAL) and occupational exposure in dairy farmers.

**Methods:**

Clinical and biological (total IgE and 21 allergen specific IgE) markers of atopy were assessed in 101 dairy farmers with PAL (DF-PAL), 85 non-farmers with PAL (NF-PAL) (both groups were prospectively included from a screening program performed between 2011 and 2015), and matched controls, i.e. 98 farmers without PAL (DF-controls) and 89 non-farming subjects without PAL (NF-controls). Occupational exposure in farmers was estimated using a validated questionnaire.

**Results:**

Prevalence of allergy history was significantly higher in DF-PAL and in NF-PAL than in controls. Polysensitization, and sensitization to seasonal and food allergens were more frequent in DF-PAL than in DF-controls, respectively: 13.8% vs 1% (adjusted odds ratio (aOR): 17.5 (2.2–134), 11.9% vs 3.1% (aOR: 4.4 (1.2–7.2) and 16.8% vs 4.1% (aOR: 5.2 (1.7–7.2)). The prevalence of atopy markers was similar between NF-PAL patients and NF-controls.

**Conclusions:**

PAL in farmers is associated with a high rate of markers of atopy, supporting atopy as a risk indicator.

Clinical trial registered with ClinicalTrials.gov (NCT02540408).

**Electronic supplementary material:**

The online version of this article (10.1186/s12931-019-1082-2) contains supplementary material, which is available to authorized users.

Chronic obstructive pulmonary disease (COPD) is a preventable and treatable disease, characterized by persistent airflow limitation, which is usually progressive [1]. The most important etiological factor for COPD is tobacco smoking, but many other factors can trigger the lung inflammation leading to COPD. Occupational exposure to vapors, gas, dust or fumes ranks high among these factors, and accounts for 20% of COPD in developed countries [2, 3]. Biological dusts may be associated with a higher risk of occupational COPD than mineral dusts [3]. Exposure to organic dusts has been shown to be associated with an increased risk of obstructive lung diseases, especially in dairy farmers [4, 5].

Although the pathophysiological traits of tobacco-related COPD are now well described, there are very few studies that have specifically assessed the pathophysiology of COPD related to organic dust exposure. Inflammatory response in tobacco-related COPD is dominated by Th1-type lymphocyte response, along with Th17 cytokine production [6] . By contrast, COPD induced by inhalation of biomass fumes could be associated with a predominantly Th2-type lymphocyte production profile, in addition to an increase in IL-4 [7].

Although there is some evidence suggesting a link between atopy and a lower FEV1 [8–10] related to organic dust exposure, especially in non-smokers, a comprehensive study of atopic response and the presence of PAL in dairy farmers using non-PAL dairy farmers as controls, as well as in subjects with or without PAL in the general population, is lacking. Since we speculate that PAL in dairy farmers may involve IgE-mediated reactions, we aimed to analyze the relationship between PAL, clinical and biological markers of atopy in farming and non-farming subjects.

## Methods

### Study design and subjects

Data for this study were collected as part of the BALISTIC project (COPD in dairy farmers: screening, characterization and constitution of a cohort; ClinicalTrials.gov Identifier: NCT02540408) (see additional file 1) [11].

A diagnosis of PAL was retained when the FEV1/FVC ratio post-bronchodilator was less than 0.70. PAL patients were rated either as stage 1 (i.e., FEV1 was > 80% of the predicted value) or stage 2+ (i.e., FEV1 was ≤80% of the predicted value). Spirometry was considered normal when the FEV1/FVC ratio was > 0.70 and FEV1 was > 80% of the predicted value before bronchodilator administration. Predicted values were based on the GLI equations [12].

All subjects in whom PAL was detected during the “screening phase” were invited to participate in the “characterization” phase of the study. Those who accepted to participate in this “characterization” phase were included in two subgroups, namely dairy farmers with PAL (DF-PAL) and non-farmers with PAL (NF-PAL). Subjects with normal spirometry who had participated in the “screening” phase of the study were frequency matched with the PAL subjects in terms of age, body mass index (BMI), tobacco smoking (in pack-years) and sex; these subjects with normal spirometry constituted 2 additional subgroups, namely, dairy farmers without PAL (DF-Controls) and non-dairy farmers without PAL (NF-Controls). Ethical approval was received from the local Ethics Committee (CPP Est; 11/617), and written consent was obtained from all subjects.

### Procedures

During the “characterization” hospital visit, subjects had a second spirometry test performed by a physician specialized in physiology, as well as several standardized questionnaires and allergological examinations. The medical questionnaire was an adapted French translation of the long version of the European Community Respiratory Health Survey questionnaire [13, 14]. The occupational questionnaire was sent to the subjects 10 days before their medical examination, collected during the medical examination, and reviewed in the presence of the subject [5, 15].

Total IgE concentration in the blood and allergen-specific IgE against 21 common food and inhalant allergens were assessed. Allergen mix was performed as the first-line. If the allergen mix was positive, then allergen-specific IgE were determined.

Blood assays were performed using enzyme-linked immunosorbent assay (ELISA), ImmunoCAP® (ThermoFisher Scientific/Phadia, Uppsala, Sweden), in the Biochemistry Laboratory at the University Hospital of Besançon, France. The total and allergen-specific IgE concentration in the blood were dichotomized at the detection limit of 100 kIU/L and 0.35 kUA/L, respectively [16, 17]. A polysensitized patient was defined as a subject having at least three positive tests for allergen-specific IgE.

In addition, analyses of dust from each patient dwelling and specific patient sensitization to his environment have been previously published [18].

### Statistical analysis

Qualitative variables are presented as number and percentage, and quantitative variables as mean ± standard deviation (SD) or median and interquartile range (IQR) (Q1-Q3).

Characteristics of PAL and control subjects were compared using the Student t-test or Wilcoxon test as appropriate; and the Chi-square or Fisher’s exact test as appropriate, for quantitative and qualitative variables, respectively.

Bivariate analyses were used to compare the results of clinical and biological markers of atopy in DF-PAL vs DF-controls, and in NF-PAL vs NF controls. The association between these atopy markers and PAL in farmers and PAL in non-farmers was assessed by using logistic regressions with adjustment for age, smoking status (< 1 pack-year (reference), 1–15 pack-years and > 15 pack-years) and sex (female as reference). Odds ratios are presented using forest plots. We used different multivariate regression models to explain PAL in the whole population in order to test the relevant atopy markers with adjustment for farming status, age, sex and smoking. Odds ratios are given with 95% confidence intervals (CI).

A *p*-value < 0.05 was considered statistically significant. Statistical analyses were performed using SAS version 9.4 (SAS Institute, Inc., Cary, NC, USA).

## Results

**Fig. 1:**
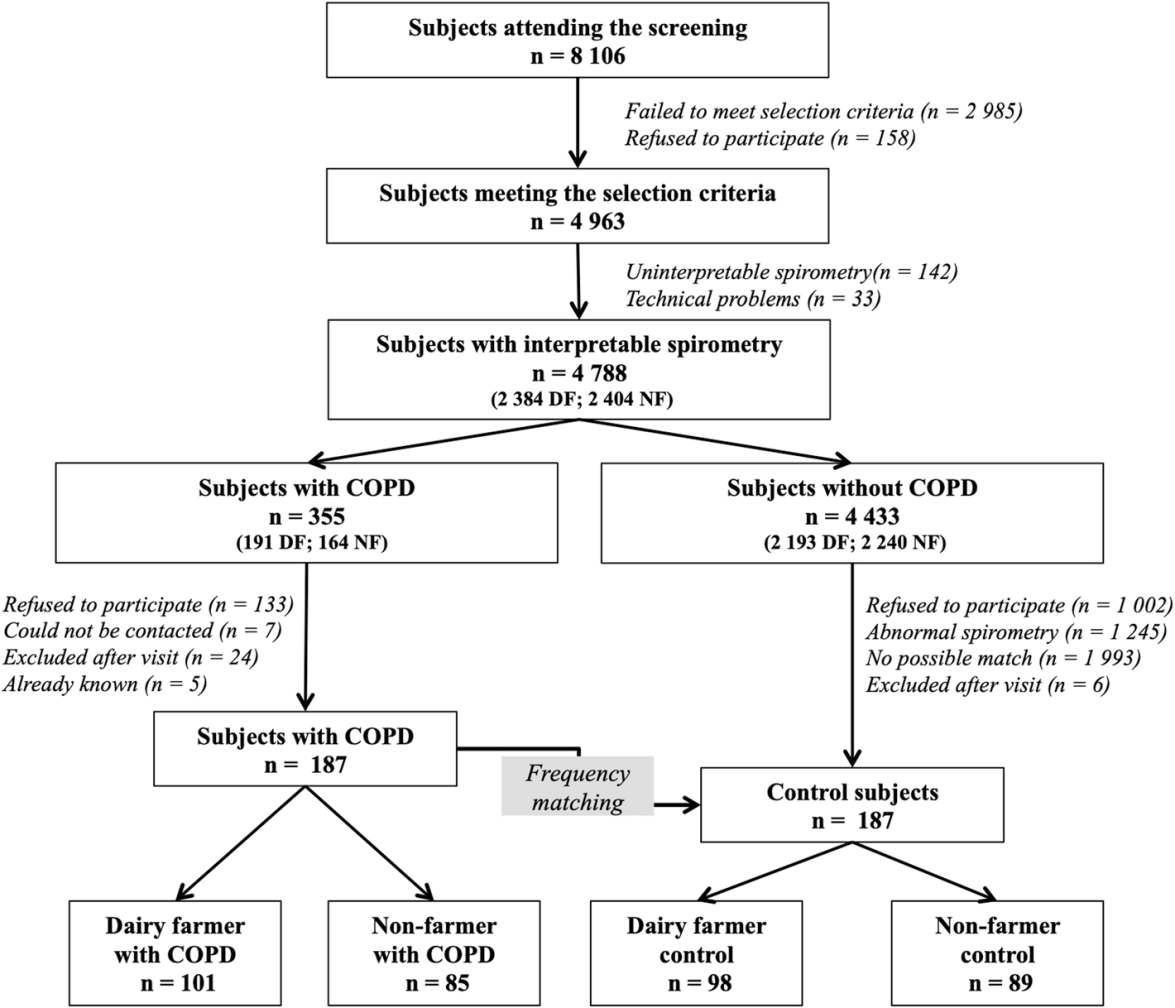
Flow chart of participants included in the study. Subjects were recruited through a screening program set up by two national health insurance organizations. Inclusion criteria in the screening programs were: men or women aged 40 to 74 years, with no history of chronic respiratory disease including asthma and hypersensitivity pneumonitis. Abbreviations: COPD: chronic obstructive lung disease; DF: dairy farmer; NF: non farmer

Among the 8106 subjects who underwent screening, 6704 were affiliated to the Mutualité Sociale Agricole (MSA), i.e. approximately 40% of the regional target population for the free health check-up. The remaining 60% of subjects were sampled to assess the representativeness of our study population. Among the 8106 subjects, 4963 met the inclusion criteria for the study (60.8%). Among the 2384 farmers with interpretable spirometry, 191 (8.01%) suffered from PAL. In total, 355 patients with PAL (191 dairy farmers and 164 non-farmers) and 3188 patients with normal spirometry were identified. 2 hundred and 10 PAL patients and 193 non-PAL matched controls agreed to take part in the “characterization” phase of the study during which 30 subjects had to be excluded, mainly owing to the existence of a history of asthma or to occupational exposure other than dairy farming. Finally, a total of 373 subjects were included: 101 DF-PAL, 98 DF-Controls, 85 NF-PAL and 89 NF-Controls (Table 1 and Fig. 1).

**Table 1:** Characteristics of the study patients (*n* = 373)

	DF- COPD	DF-Control	*p*-value	NF- COPD	NF-Control	p-value
	*n* = 101	*n* = 98		*n* = 85	*n* = 89	
Age, years	60.3 ± 9.1	59.3 ± 8.9	NS	61.4 ± 7.6	60.2 ± 6.5	NS
Men	86 (85.1)	86 (87.8)	NS	66 (77.6)	65 (73.0)	NS
BMI	26.5 ± 4.1	27.0 ± 4.1	NS	26.1 ± 4.1	26.8 ± 3.8	NS
Smoking status			NS			NS
Non-smoker	48 (47.5)**	54 (55.1)		7 (8.2)	12 (13.5)	
Former-smoker	33 (32.7)**	32 (32.7)		36 (42.4)	42 (47.2)	
Currentsmoker	20 (19.8)**	12 (12.2)		42 (49.4)	35 (39.3)	
Smoking pack years			NS			NS
< 1	49 (48.5)**	55 (56.1)		7 (8.2)	13 (14.6)	
1–15	18 (17.8)**	21 (21.4)		13 (15.3)	15 (16.9)	
> 15	34 (33.7)**	22 (22.5)		65 (76.5)	61 (68.5)	
Exacerbation	23 (22.8)			23 (27.1)		
At least one respiratory symptom	54 (53.1)	21 (21.3)	**< 0.001**	51 (60.0)	23 (25.9)	**< 0.001**
Dyspnea, mMRC> 0	38 (38.0)	15 (15.3)	**< 0.001**	39 (45.9)	19 (21.4)	**< 0.001**
SGRQ	15.1 ± 12.9*	7.3 ± 7.2	**< 0.001**	19.4 ± 14.9	9.3 ± 9.3	**< 0.001**
FEV1 /FVC post-BD, %pred	63.0 ± 6.8*	78.5 ± 4.3	**< 0.001**	60.6 ± 8.0	79.9 ± 4.1	**< 0.001**
FEV1 post-BD, %pred;	85.2 ± 14.8*	105.5 ± 10.9	**< 0.001**	80.0 ± 16.4	108.3 ± 12.7	**< 0.001**
FVC post-BD, %pred;	105.3 ± 15.7	104.8 ± 11.4	NS	102.1 ± 14.8	105.7 ± 12.0	NS
Severity of airway obstruction, n (%)						
Stage I	44 (44)			33 (39)		
Stage II +	57 (56)			52 (61)		

Data are presented as n, n (%) or mean ± SD, unless otherwise stated

*COPD* chronic obstructive pulmonary disease, *DF-COPD* dairy farmers with COPD, *NF-COPD* non-dairy farmers with COPD, *SD* standard deviation, *BMI* body mass index, *mMRC* modified Medical Research Council, *SGRQ* St George’s Respiratory Questionnaire, *FVC* forced vital capacity, *FEV1* forced expiratory volume in 1 s

* *p* < 0.05, vs NF- COPD, ** *p* < 0.01, vs NF- COPD

Significant *P* values (< 0.05) are in bold

A total of 365 subjects affiliated to the MSA and not present at the screening were successfully contacted. They were older on average (61.9 ± 10.0 vs 60.2 ± 9.7 years, *p* = 0.0014) and more often male (72.6% vs 57.5%, *p* <  0.0001) than subjects who participated in the screening, but their smoking status was not statistically different. In addition, patients who participated in the hospital “characterization” visit were not different from those screened (PAL and non-PAL groups) in terms of smoking status, sex-ratio, spirometry (% of theoretical values), but they were younger (60.3 vs 64.2 years, *p* = 0.0047).

The main features of the four groups are summarized in Table 1. Both PAL groups were composed mainly of mild PAL with preserved FEV1. The proportion of current-smokers and former-smokers was lower among farmers than in non-farmers. The proportion of men was higher among farmers than among non-farmers, but the difference was not statistically significant.

The mean level of bronchodilation during the bronchodilation test was similar in DF-PAL and NF-PAL: 8.2% ± 8.2 vs 7.8% ± 7.5, *p* = 0.7202, respectively.

**Fig. 2:**
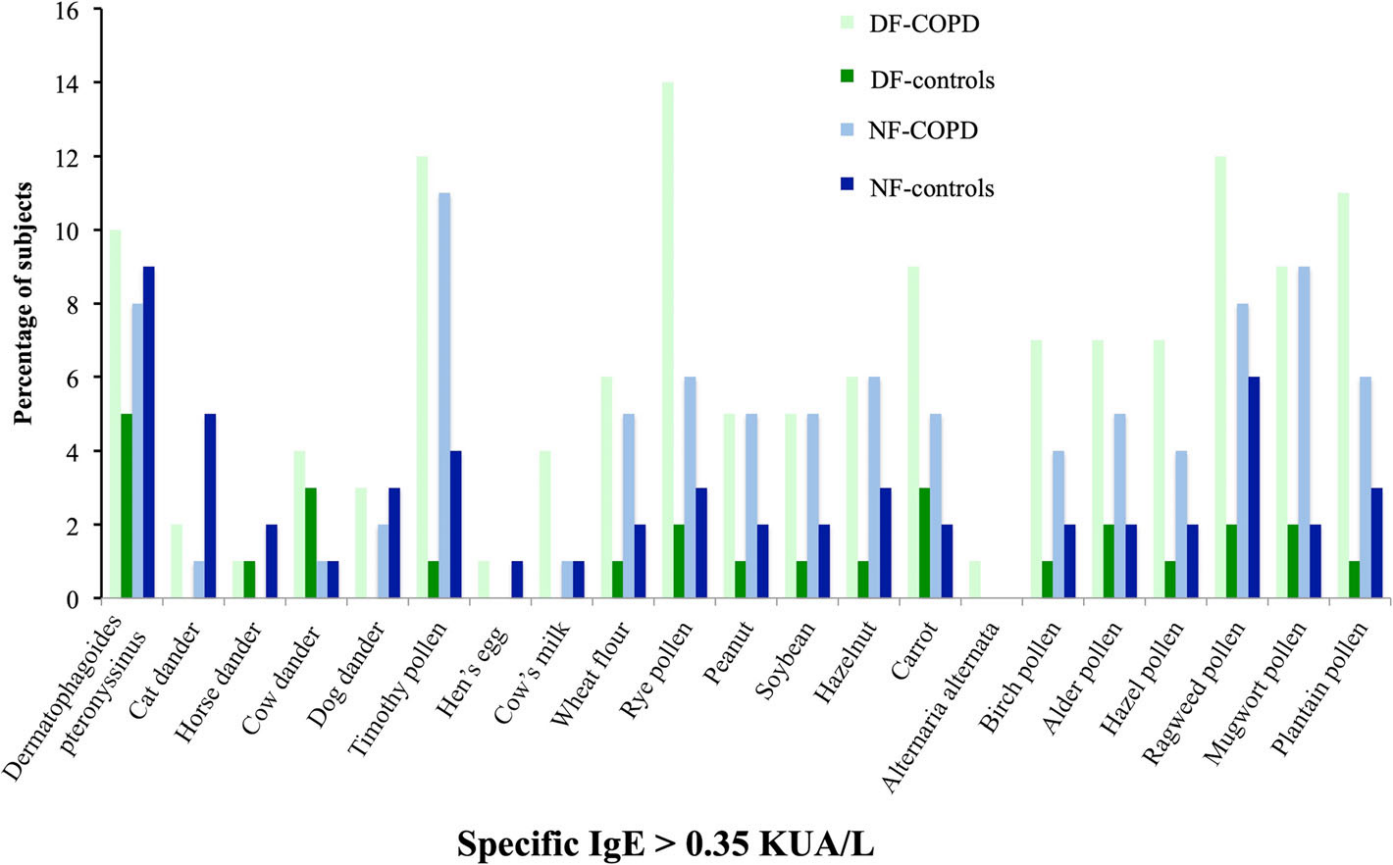
Frequency histogram of specific IgE among dairy-farmers and non-dairy-farmers’ patients COPD: chronic obstructive pulmonary disease; DF-COPD: dairy farmers with COPD; DF-control: dairy farmers in control group; NF- COPD: COPD in non-dairy farmers; NF-control: non-dairy farmers in control group.

The atopy markers among the groups are shown in Table 2. There was a statistically significant relation between a self-reported personal history of allergy and PAL. In dairy farmers, the frequency of sensitizations to food allergens and seasonal inhalant allergens was significantly higher in DF-PAL. No significant relationship was found between atopy markers and PAL in non-farmers. Overall, specific IgEs were higher in the DF-PAL group than in the DF-controls for all allergens (Fig. 2). No significant relationship was found between occupational exposure in dairy farming and PAL in farmers (additional file 2).

**Table 2:** History of atopy, specific IgE level and total IgE amount among dairy-farmers and non-dairy-farmers

	DF-COPD	DF-Control	p-value	NF- COPD	NF-Control	p-value
	n = 101	n = 98		n = 85	n = 89	
Personal history of hay fever, %	8.9	10.2	NS	14.1	12.8	NS
Personal history of atopic dermatitis, %	5.0	5.1	NS	9.6	4.6	NS
Personal history of allergy, %	60.0	44.3	**0.0277**	70.2	52.8	**0.0187**
Family history of allergy, %	46.4	32.9	NS	42.6	41.1	NS
Wheezing, %	31.7	6.3	**< 0.0001**	29.4	11.2	**0.0028**
Total IgE amount						
median (Q1-Q3)	40 (17–125)	37 (18–81)	NS	42 (13–114)	28 (15–75)	NS
> 100 IU/mL, %	30.3	24.7	NS	31.7	17.1	**0.0256**
Positive specific IgE (> 0.35 kUA/L)						
At least one, %	21.8	14.4	NS	28.2	23.9	NS
Food allergens, %	16.8*	4.1	**0.0037**	7.1	5.6	NS
Perennial inhalant allergens, %	10.9	9.3	NS	10.6	12.4	NS
Seasonal inhalant allergens, %	11.9	3.1	**0.0195**	21.2	11.4	NS
Polysensitized (at least 3 IgE)	13.8	1.0	**0.0006**	8.2	6.7	NS

*COPD* chronic obstructive pulmonary disease, *DF-COPD* dairy farmers with COPD, *NF-COPD* non-dairy farmers with COPD, *SD* standard deviation, allergy was self reported, history of allergy covered nasal allergies including hay fever, eczema or any kind of skin allergy, or allergy to insect stings or bites. * p < 0.05, vs NF- COPD

Significant *P* values (< 0.05) are in bold

**Fig. 3:**
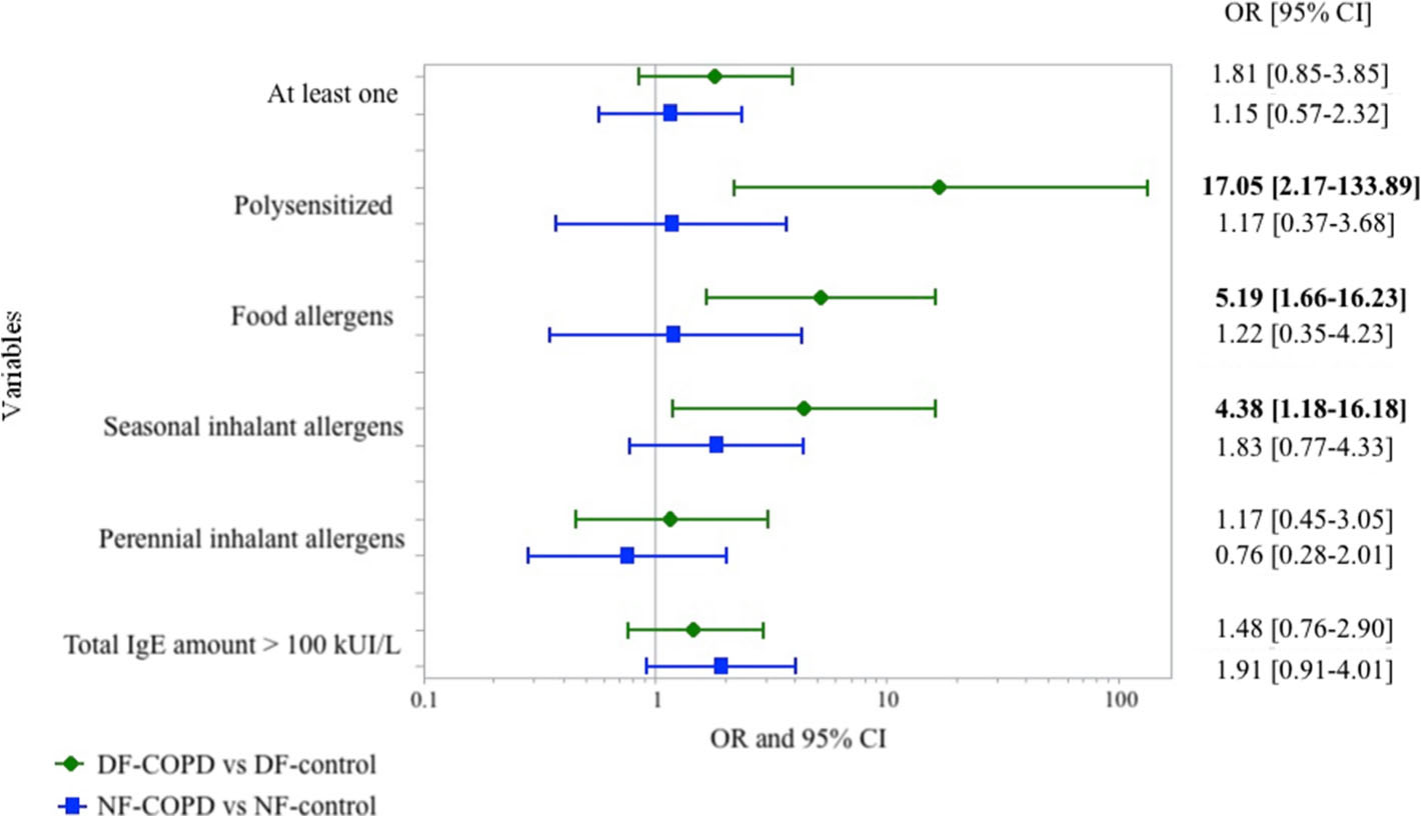
Comparison of odds ratios for markers of atopy among dairy-farmers and non-dairy-farmers (reference groups are patients without chronic obstructive pulmonary disease (COPD). All ORs were adjusted for age (continuous), gender (reference: female) and pack-years (< 1, 1–15, > 15)

As there were slight, albeit non-statistically significant differences between PAL groups and their controls in terms of age, gender and smoking, we adjusted for these variables when comparing the distribution of biological markers of atopy in PAL and controls separately in farmers and in non-farmers (Fig. 3). Results showed a higher prevalence of IgE sensitization only in PAL in farmers (Fig. 3).

Then, we constructed three different models to determine factors associated with PAL in the whole population. After adjustment for potential confounders, PAL was mainly explained by the presence of biological atopy markers and smoking (> 15 pack-years) (Table 3). The use of the same models replacing PAL by FEV1 as the outcome variable showed the same results. Models using perennial inhalant allergens or at least one IgE were not relevant. Regarding atopy markers, we did not find an association with occupational exposure after adjustment except for seasonal inhalant allergens which were inversely associated with diary farming (additional file 3).

**Table 3:** Adjusted odds ratios for chronic obstructive pulmonary disease in the whole population considering three tested indicators of atopy

	COPD		
	OR	95% CI	p-value
Model 1			
Food allergens; ref.: no	**2.71**	**1.23–6.46**	**0.0176**
Nasal allergies; ref.: no	**2.17**	**1.34–3.56**	**0.0019**
Farmers; ref.: non-farmer	1.41	0.86–2.31	0.1725
Age; continuous	1.02	0.99–1.05	0.1259
Sex; ref.:female	1.06	0.61–1.84	0.8370
Pack-years; ref. < 1	1		
1–15	1.13	0.60–2.13	0.7039
> 15	1.69	0.98–2.92	0.0599
Model 2			
Seasonal inhalant allergens; ref.: no	**2.57**	**1.29–5.39**	**0.0091**
Nasal allergies; ref.: no	**2.21**	**1.36–3.63**	**0.0015**
Farmers; ref.: non-farmer	1.57	0.96–2.60	0.0746
Age; continuous	1.02	0.99–1.05	0.1681
Sex; ref.:female	1.04	0.60–1.82	0.8861
Pack-years; ref. < 1	1		
1–15	1.07	0.57–2.01	0.8352
> 15	1.64	0.96–2.85	0.0737
Model 3			
Polysensitized (at least 3 IgE); ref.: no	**3.45**	**1.46–9.12**	**0.0073**
Nasal allergies; ref.: no	**2.25**	**1.38–3.70**	**0.0011**
Farmers; ref.: non-farmer	1.44	0.89–2.37	0.1427
Age; continuous	1.02	0.99–1.05	0.1081
Sex; ref.:female	1.15	0.66–2.02	0.6178
Pack-years; ref. < 1	1		
1–15	1.14	0.61–2.14	0.6849
> 15	1.67	0.97–2.89	0.0669

Logistic regressions of chronic obstructive pulmonary disease (COPD) for atopy markers and covariates that are known to be independent risk factors for COPD; models using perennial inhalant allergens and at least one IgE like indicators of atopy have shown no significant difference

Significant *P* values (< 0.05) are in bold

Finally, non-smoker DF-PAL were compared to smoker DF-PAL (current and former) for age, sex, clinical and biological atopy markers. No differences were identified.

## Discussion

In this study, the frequency of atopy markers was higher in PAL-groups than among controls, but the difference was driven by a significantly higher rate among dairy farmers only. Our study suggests that IgE-mediated reactions may play a role in PAL development in dairy farming. This hypothesis is reinforced by the fact that in farmers, there was no relationship between occupational exposure and either PAL or atopy markers. Indeed, data issued from the same cohort showed similar exposure pattern among dairy farmers and therefore exposure could not explain the occurrence of COPD [18]. This may suggest a key role of individual factors in the genesis of COPD in dairy farming.

Since atopy is not a usual characteristic for subjects with COPD, this supports the hypothesis that COPD in dairy farmers is different than COPD associated with tobacco smoking. Our results suggest that IgE-mediated reactions (therefore possibly a Th2 cytokine production profile) are involved in the development of COPD in dairy farming. COPD due to tobacco smoking is dominated by Th1-type lymphocyte response [6] and is associated with Th17 cytokine production [7]. Conversely, COPD induced by exposure to biomass smoke might be associated with a predominantly Th2-type lymphocyte production profile, as in asthma, and an increase of IL-4 [7, 19]. Moreover, emphysema is rarely found in this type of COPD [7]. COPD in dairy farming could be close to COPD induced by exposure to biomass smoke [19].

The involvement of atopy in COPD has never been extensively studied, to the best of our knowledge. Eduard et al. reported that Norwegian farmers with atopy had a significantly lower FEV1 than non-atopic counterparts [8]. However, in their study, no bronchodilation test was performed, and the relationship between FEV1 and atopy disappeared after the exclusion of asthmatics. In addition, atopy was defined as a positive ImmunoCap® result [8]. In our study, measurements of allergen-specific IgEs against 21 common food and inhalant allergens were performed. Another study in farmers showed that a total IgE concentration > 180 kIU/L was associated with lower FEV1 [20]. More recently, Cushen et al. observed that among non-smoker Irish farmers, those with lung obstruction more often had histories of hay fever or allergies [21]. However, according to the authors, the presence of asthmatics at least partly explains these results. Nonetheless, in previous studies, no significant relationships emerged between atopy markers and COPD. Ours seems to be the first study showing a strong link between markers of atopy and COPD. In contrast with previous studies performed in the Franche-Comté region [5], we did not find any link between the level or type of occupational exposure and agricultural COPD prevalence. The most likely explanation is a progressive homogenization of occupational exposure, with modernization of dairy farms over time.

The associations observed in our study between COPD in dairy farmers and IgE sensitization concerns mainly seasonal allergens. These results are in line with the excess of prior history of personal allergy, and the higher degree of wheezing observed in DF-COPD. However, the absence of any difference between groups of farmers regarding hay fever is unusual (Table 2). This is possibly due to the fact that we excluded asthmatics and consequently, allergic subjects. The relationship between COPD in farmers and food allergens may be partially explained by cross-reactions between food and seasonal inhalant allergens. It is noteworthy that the protection against allergy conferred by dairy farming observed in the large PASTURE European birth cohort that included farmers living in the same region as the present study, also concerned seasonal and food allergens, but not perennial ones [22]. Dairy farming may be associated with a reduced risk of lung cancer [23] and is known to protect against allergy in children, but possibly also in adults [9]. Therefore, those who still develop atopy even with an early-life farm exposure are those who may be at risk of COPD. Besides, this occupational environment is also known to generate COPD, especially in regions where organics dusts and microorganisms constitute the main exposure [4, 5]. We can therefore hypothesize that this exposure in atopic subjects causes a bronchial disease different from asthma, which is characterized by a FEV1/FVC ratio after bronchodilation < 0.7. Analyses of cytokine measurements as well as IgE and IgG-mediated reactions against microorganisms present in the home of the studied subjects are planned and could be of substantial interest in understanding the mechanisms of COPD in dairy farmers.

Our study includes limitations inherent to a large screening program. Only subjects who accepted to undergo a health check-up were included in the COPD screening program. In addition, only half the subjects who had COPD detected by screening accepted to attend the hospital visit for “characterization”. It has previously been reported that among all subjects who were invited to health check-ups organized by the French agricultural health insurance system, those who attend these check-ups and those who do not have different health characteristics [4]. It is therefore possible that our population was not representative of the whole population of dairy farmers (target population) regarding health status. Our studied population, however, is not different from the target population in terms of smoking habits. There are significant differences for age and sex ratio, but neither of these factors were associated with atopy markers in the present analysis. Finally, our outcomes can only be considered robust if we are sure that the diagnosis of COPD was accurate, and that patients with asthma were truly excluded. All reasonable precautions were taken to verify the diagnosis of COPD. Spirometries were performed by trained nurses during the “screening” phase, followed by diagnostic confirmation in the Department of Respiratory Medicine & Physiology of our university hospital. Subjects with self-reported asthma were excluded. Therefore, we can rule out an asthma-COPD-overlap syndrome, which would have been more frequent in DF-COPD. Farmer’s lung disease, another possible differential diagnosis in our farming region, was also ruled out by a question in the medical questionnaire and thanks to local expertise in this disease [24].

## Conclusion

Dairy farmers with COPD are more polysensitized than non-farming patients with COPD, and more polysensitized, sensitized to food and seasonal inhalant allergens than non-COPD subjects. These results expand the hypothesis that atopy may be a risk indicator for COPD in dairy farming, distinguishing it from COPD associated with tobacco smoking.

## Additional files

Additional file 1: Supplementary methods (DOCX 25 kb)

Additional file 2: Occupational characteristics of dairy farming COPD and controls. (DOCX 20 kb)

Additional file 3: Adjusted odds ratios for markers of atopy in the whole population considering three tested indicators (DOCX 17 kb)

## Data Availability

All data generated or analysed during this study are included in this published article and its supplementary information files.

## Abbreviations

BMI: Body mass index
CI: Confidence intervals
COPD: Chronic obstructive pulmonary disease
DF: Dairy farmers
ELISA: Enzyme-linked immunosorbent assay
FEV1: Forced expiratory volume in 1 s
FVC: Forced vital capacity
IQR: Interquartile range
mMRC: modified Medical Research Council
MSA: Mutualité Sociale Agricole
NF: Non-dairy farmers
SD: Standard deviation
SGRQ: St George’s Respiratory Questionnaire.

## References

[1] Vogelmeier CF, Criner GJ, Martinez FJ (2017). Global strategy for the diagnosis, management, and prevention of chronic obstructive lung disease 2017 report. GOLD executive summary. Am J Respir Crit Care Med.

[2] Omland O, Wurtz ET, Aasen TB (2014). Occupational chronic obstructive pulmonary disease: a systematic literature review. Scand J Work Environ Health.

[3] Sadhra S, Kurmi OP, Sadhra SS (2017). Occupational COPD and job exposure matrices: a systematic review and meta-analysis. Int J Chron Obstruct Pulmon Dis.

[4] Guillien A, Puyraveau M, Soumagne T (2016). Prevalence and risk factors for COPD in farmers: a cross-sectional controlled study. Eur Respir J.

[5] Marescaux A, Degano B, Soumagne T (2016). Impact of farm modernity on the prevalence of chronic obstructive pulmonary disease in dairy farmers. Occup Environ Med.

[6] Barnes PJ (2016). Inflammatory mechanisms in patients with chronic obstructive pulmonary disease. J Allergy Clin Immunol.

[7] Solleiro-Villavicencio H, Quintana-Carrillo R, Falfan-Valencia R (2015). Chronic obstructive pulmonary disease induced by exposure to biomass smoke is associated with a Th2 cytokine production profile. Clin Immunol.

[8] Eduard W, Pearce N, Douwes J (2009). Chronic bronchitis, COPD, and lung function in farmers: the role of biological agents. Chest..

[9] House JS, Wyss AB, Hoppin JA (2017). Early-life farm exposures and adult asthma and atopy in the agricultural lung health study. J Allergy Clin Immunol.

[10] Lluis A, Depner M, Gaugler B (2014). Increased regulatory T-cell numbers are associated with farm milk exposure and lower atopic sensitization and asthma in childhood. J Allergy Clin Immunol.

[11] Degano B, Bouhaddi M, Laplante JJ (2012). COPD in dairy farmers: screening, characterization and constitution of a cohort. The BALISTIC study. Rev Mal Respir.

[12] Quanjer PH, Stanojevic S, Cole TJ (2012). Multi-ethnic reference values for spirometry for the 3-95-yr age range: the global lung function 2012 equations. Eur Respir J.

[13] Dalphin JC, Dubiez A, Monnet E (1998). Prevalence of asthma and respiratory symptoms in dairy farmers in the French province of the Doubs. Am J Respir Crit Care Med.

[14] Burney PG, Luczynska C, Chinn S (1994). The European Community respiratory health survey. Eur Respir J.

[15] Mauny F, Polio JC, Monnet E (1997). Longitudinal study of respiratory health in dairy farmers: influence of artificial barn fodder drying. Eur Respir J.

[16] Fernandez C, Cardenas R, Martin D (2007). Analysis of skin testing and serum-specific immunoglobulin E to predict airway reactivity to cat allergens. Clin Exp Allergy.

[17] Kerkhof M, Dubois AE, Postma DS (2003). Role and interpretation of total serum IgE measurements in the diagnosis of allergic airway disease in adults. Allergy..

[18] Barrera Coralie, Rocchi Steffi, Degano Bruno, Soumagne Thibaud, Laurent Lucie, Bellanger Anne-Pauline, Laplante Jean-Jacques, Millon Laurence, Dalphin Jean-Charles, Reboux Gabriel (2018). Microbial exposure to dairy farmers’ dwellings and COPD occurrence. International Journal of Environmental Health Research.

[19] Olloquequi J, Jaime S, Parra V, et al. Comparative analysis of COPD associated with tobacco smoking, biomass smoke exposure or both. Respir Res. 2018;19(13). 10.1186/s12931-018-0718-y. 10.1186/s12931-018-0718-y PMC5774164 29347936

[20] Westeel V, Julien S, De Champs C (2000). Relationships of immunoglobulins E and G sensitization to respiratory function in dairy farmers. Eur Respir J.

[21] Cushen B, Sulaiman I, Donoghue N (2016). High prevalence of obstructive lung disease in non-smoking farmers: the Irish farmers lung health study. Respir Med.

[22] Ege MJ, Herzum I, Buchele G (2008). Prenatal exposure to a farm environment modifies atopic sensitization at birth. J Allergy Clin Immunol.

[23] Tual S, Lemarchand C, Boulanger M (2017). Exposure to farm animals and risk of lung Cancer in the AGRICAN cohort. Am J Epidemiol.

[24] Depierre A, Dalphin JC, Pernet D (1988). Epidemiological study of farmer's lung in five districts of the French Doubs province. Thorax..

