# Supplementary material for: Is atopy a risk indicator of chronic obstructive pulmonary disease in dairy farmers?
Source: Respir Res. 2019 Jun 17;20:124. doi: 10.1186/s12931-019-1082-2 (PMC6580567; doi:10.1186/s12931-019-1082-2)
Supplement: Supplementary file 1 — Supplementary methods (DOCX 25 kb) [file 12931_2019_1082_MOESM1_ESM.docx]

Additional file 1: Supplementary methods

**METHODS**

**Screening program**

The BALISTIC project (COPD in dairy farmers: screening, characterization and constitution of a cohort; ClinicalTrials.gov Identifier: NCT02540408) which was conducted from 2011 to 2015 at the University Hospital of Besançon in collaboration with the French national social security system for agricultural workers (Mutualité Sociale Agricole, MSA) and the federation of community health practices of Franche-Comté (Fédération des Maisons de Santé Comtoises, FeMaSaC). This study was set up to assess the prevalence, and specific characteristics of COPD in dairy farmers (COPD secondary to organic dust exposure) in comparison with COPD in patients without any occupational exposure, and compared to matched controls without COPD [1].

COPD patients and controls were recruited through a screening program (“screening” phase of the BALISTIC study) in two branches of the social security. Inclusion criteria in the screening programs were: men or women aged 40 to 74 years, with no history of chronic respiratory disease including asthma, hypersensitivity pneumonitis, and who were either a dairy farmer (“dairy farmers” subgroups) or unexposed to any occupational hazard associated with COPD (“non-farmers” subgroups). COPD screening was proposed to all invited subjects who attended the health check-up organized by the MSA or were invited by the general practitioners (GP) of the FeMaSaC and who fulfilled the inclusion criteria. A random sample of 5% of the non-respondent subjects (i.e. farmers who did not attend the health check-up) was drawn to compare participants and non-participants with regard to age, sex and smoking habits.

For the screening, spirometry was performed as previously described [2]. Spirometry outcomes included forced expiratory volume in 1 second (FEV1) and forced vital capacity (FVC). A bronchodilation test was applied when the FEV1/FVC ratio was less than 0.70 [3]. Predicted values were based on the GLI equations [4].

**Outcome definitions**

A never-smoker was defined as a subject having smoked less than one cigarette, one cigar or one pipe a day for one year. An ex-smoker was defined as a subject having stopped smoking for at least one month before completing the questionnaires. Chronic bronchitis was defined as cough and daily sputum three months a year for two consecutive years.

1. Degano B, Bouhaddi M, Laplante JJ, et al. [COPD in dairy farmers: screening, characterization and constitution of a cohort. The BALISTIC study]. Rev Mal Respir. 2012;29:1149-56.<http://dx.doi.org/10.1016/j.rmr.2012.08.007>.

2. Guillien A, Puyraveau M, Soumagne T, et al. Prevalence and risk factors for COPD in farmers: a cross-sectional controlled study. Eur Respir J. 2015. <http://dx.doi.org/10.1183/13993003.00153-2015>.

3. Miller MR, Hankinson J, Brusasco V, et al. Standardisation of spirometry. Eur Respir J. 2005;26:319-38. <http://dx.doi.org/10.1183/09031936.05.00034805>.

4. Quanjer PH, Stanojevic S, Cole TJ, et al. Multi-ethnic reference values for spirometry for the 3-95-yr age range: the global lung function 2012 equations. Eur Respir J. 2012;40:1324-43. <http://dx.doi.org/10.1183/09031936.00080312>.
