# Supplementary material for: Is atopy a risk indicator of chronic obstructive pulmonary disease in dairy farmers?
Source: Respir Res. 2019 Jun 17;20:124. doi: 10.1186/s12931-019-1082-2 (PMC6580567; doi:10.1186/s12931-019-1082-2)
Supplement: Supplementary file 2 — Occupational characteristics of dairy farming COPD and controls. (DOCX 20 kb) [file 12931_2019_1082_MOESM2_ESM.docx]

Additional file 2: Occupational characteristics of dairy farming COPD and controls.

|  | | DF-COPD | DF-Control | p |
| --- | --- | --- | --- | --- |
|  |  | n = 101 | n = 98 |  |
| Activity level | |  |  | NS |
|  | Retired, no longer working on a farm | 28 (29) | 25 (26) |  |
|  | Retired, still working on a farm | 22 (23) | 22 (22) |  |
|  | Active | 47 (47) | 51 (52) |  |
| Farm’s characteristics for at least 10 years (presence of) | | | | |
|  | Separation between house and cowshed | 74 (73) | 71 (72) | NS |
|  | Loading grippers | 28 (28) | 29 (30) | NS |
|  | Food grippers | 15 (15) | 17 (18) | NS |
|  | Straw blower | 21 (21) | 23 (24) | NS |
|  | Central corridor | 75 (74) | 80 (82) | NS |
|  | Loose housing system | 50 (51) | 55 (59) | NS |
|  | Ventilation | 44 (44) | 37 (38) | NS |
|  | Milking parlour | 54 (53) | 59 (60) | NS |
|  | Barn drying system | 18 (18) | 21 (21) | NS |
| Size of the farm during the past 10 years of work | | | | |
|  | Total size, hectares | 95±56 | 97±58 | NS |
|  | Size of fodder lands, hectares | 52±34 | 57±34 | NS |
|  | Size of cereal production, hectares | 24±35 | 29±42 | NS |
|  | Number of cattle | 109±69 | 107±72 | NS |
|  | Number of cows | 43±21 | 44±23 | NS |

Data are presented as n, n (%) or mean ± SD, unless otherwise stated.

COPD: chronic obstructive pulmonary disease; DF-COPD: dairy farmers with COPD; DF-Control: dairy farmers in control group.
