# Supplementary material for: Is atopy a risk indicator of chronic obstructive pulmonary disease in dairy farmers?
Source: Respir Res. 2019 Jun 17;20:124. doi: 10.1186/s12931-019-1082-2 (PMC6580567; doi:10.1186/s12931-019-1082-2)
Supplement: Supplementary file 3 — Adjusted odds ratios for markers of atopy in the whole population considering three tested indicators (DOCX 17 kb) [file 12931_2019_1082_MOESM3_ESM.docx]

|  | **COPD** | | |
| --- | --- | --- | --- |
|  | OR | 95% CI | p-value |
| **Model 1** (Food allergens) | | | |
| Farmers; ref: non-farmer | 1.41 | 0.59 - 3.51 | 0.4383 |
| PAL; ref: no | **2.90** | **1.33 - 6.85** | **0.0099** |
| Age; continuous | 0.98 | 0.94 - 1.02 | 0.3367 |
| Sex; ref:female | 2.11 | 0.69 - 9.91 | 0.2406 |
| Pack-years; ref<1 | 1 |  |  |
| 1-15 | 0.73 | 0.22 - 2.13 | 0.5846 |
| >15 | 0.77 | 0.30 - 1.94 | 0.5769 |
| **Model 2 (**Seasonal inhalant allergens) | | | |
| Farmers; ref: non-farmer | **0.40** | **0.18 - 0.85** | **0.0201** |
| PAL; ref: no | **2.64** | **1.34 - 5.47** | **0.0066** |
| Age; continuous | 1.00 | 0.96 - 1.05 | 0.8178 |
| Sex; ref:female | 2.13 | 0.85 - 6.53 | 0.1376 |
| Pack-years; ref<1 | 1 |  |  |
| 1-15 | 1.40 | 0.48 - 3.99 | 0.5258 |
| >15 | 1.11 | 0.45 - 2.90 | 0.8179 |
| **Model 3 (**Polysensitized (at least 3 IgE) | | | |
| Farmers; ref: non-farmer | 0.99 | 0.39 - 2.52 | 0.9926 |
| PAL; ref: no | **3.47** | **1.48 - 9.10** | **0.0064** |
| Age; continuous | 0.96 | 0.92 - 1.01 | 0.1325 |
| Sex; ref:female | 0.59 | 0.24 - 1.62 | 0.2761 |
| Pack-years; ref<1 | 1 |  |  |
| 1-15 | 0.57 | 0.12 - 2.01 | 0.4123 |
| >15 | 0.94 | 0.35 - 2.55 | 0.8979 |

Additional Table 3: Adjusted odds ratios for markers of atopy in the whole population considering three tested indicators
